# The impact of 1.5 °C and 2.0 °C global warming on global maize production and trade

**DOI:** 10.1038/s41598-022-22228-7

**Published:** 2022-10-14

**Authors:** Kuo Li, Jie Pan, Wei Xiong, Wei Xie, Tariq Ali

**Affiliations:** 1 Institute of Environment and Sustainable Development in Agriculture, Chinese Academy of Agricultural Sciences, Beijing, 100081 China; 2 International Maize and Wheat Improvement Center, Texcoco, Mexico; 3 Peking University, Beijing, China

**Keywords:** Ecology, Plant sciences, Climate sciences, Environmental sciences, Natural hazards

## Abstract

Climate change is becoming more and more remarkable which has an obvious impact on crop yields all over the world. Future climate scenario data was simulated by 5 climate models recommended by ISI-MIP under 4 RCP scenarios, in which the approximate scenarios with global warming by 1.5 °C and 2 °C were selected. Applying DSSAT and GTAP models, the per unit yield changes of maize in the world under global warming by 1.5 °C and 2.0 °C were analyzed and the market prices of maize at national and global levels were simulated. The results showed that, the risk of maize yield reduction under 2.0 °C scenario was much more serious than 1.5 °C scenario; the ratios of yield changes were separately 0.18% and − 10.8% under 1.5 °C and 2.0 °C scenarios. The reduction trend of total maize production is obvious in the top five countries and the main producing regions of the world, especially under the 2.0 °C scenario. The market price of maize would increase by around 0.7% and 3.4% under 1.5 °C and 2.0 °C scenarios. With the quickly increasing population in the world, it is urgent for all countries to pay enough attention to the risk of maize yield and take actions of mitigation and adaptation to climate change.

## Introduction

In the past hundred years, the global climate has experienced great changes^1–4^. According to the sixth assessment report of IPCC, the global average surface temperature increased by 1.09 °C between 1850 and 2020, and almost all regions in the world experienced surface warming^5^. Due to global warming, the extreme climate events become more and more frequent, and the ecological environment problems caused by climate change are more and more serious, which restrict the sustainable development of human society and health^6–10^. Global warming has gradually changed from a scientific issue to a major social issue of common concern to governments and people of all countries^11–13^. In 2016, nearly 200 parties of the United Nations Framework Convention on climate change reached the Paris Agreement at the climate change conference in Paris^14^. Paris Agreement has indicated that it is urgent to hold the increase in global average temperature well below 2.0 °C above pre-industrial levels and pursue efforts to limit the temperature increase to 1.5 °C above pre-industrial levels.

Faced with climate change, agriculture is the most vulnerable sector, which will experience the largest negative impacts from climatic change and lead to more serious food security in the whole world^15–20^. Meanwhile, global production losses might lead to price shocks and trigger export restrictions^21–24^; an increasingly interconnected global food system^25,26^ and the projected fragility of the global food production system due to climatic change further exacerbate the threats to food security in the worldwide^27–29^. So, the impacts of climate changes on crop yields and prices have been of highly concerned. Numerous studies have revealed that the warming trend has negative impact on crop yields and global trade in most regions all over the world^30–32^. There are three main methods for impacts assessment of climate change on crops, including environment-controlled experiments, statistical regression analysis and model simulations^17,33^. Environment-controlled experiments are designed to observe the influence of climate factors on crops, such as drought, flood, heat stress, cold damage, elevated CO_2_ concentration, through which the impact mechanism of climate change on crops would be revealed and established^23,34,35^. Crop models and trade models are applied to simulate the response of crop yield and market price under climate change, based on process-based crop growth in daily time steps, either in selected field sites or in selected regions^36–39^. The statistical regression analysis usually explores the relationship between historical crop yields and meteorological records in different sites or counties to establish regression functions for crop responses predictions^40–43^. These researches have documented that crop yield and price would be threatened much more seriously by global warming, especially due to the increasing trend of frequency and intensity of climate extreme events in the future.

Although, so far there are plenty of research on the impacts of global warming by 1.5 °C temperature, including the impacts comparison of global warming by 1.5 °C versus 2.0 °C^44^. It is necessary to do more quantitative impacts assessments of global warming by 1.5 °C and 2.0 °C on crops yield and market price to address research gaps and support the requirement of the scientific community and governments. In this paper, the future climate situations were selected and analyzed which are the approximate scenarios with global warming by 1.5 °C and 2.0 °C, based on the simulation results from 5 climate models recommended by ISI-MIP under 4 RCP scenarios. Then the per unit yield changes of maize all over the world under global warming by 1.5 °C and 2.0 °C were analyzed and the spatial distributions of changes in maize yield were revealed relative to the baseline from 1985 to 2006, applying crop model DSSAT (Decision Support System for Agrotechnology Transfer). Next, we examine the effects of the resulting maize production shocks in different countries; the market price of maize is simulated using GTAP to reveal the impacts of climate change on global crop trade. Finally, the future trend of maize yield and market price in the main breadbasket is assessed and the adaptation suggestions are put forward for maize cultivation.

## Materials and methods

### Data processing

In this study, historical daily weather data (1986–2005) are from the AgMERRA dataset. AgMERRA is a post-processing of the NASA Modern-Era Retrospective Analysis for Research and Applications (MERRA) data. The dataset is proved to be suitable for agricultural modelling and features consistent, daily time-series data^45^.

For future (2020–2099), the original climate scenario data (Table 1) were extracted from output archives of five ESMs (including GFDL-ESM2M, HadGEM2-ES, IPSL-CM5A-LR, MIROC-ESM-CHEM and NorESM1-M) under four RCPs (RCP2.6, RCP4.5, RCP6.0, RCP8.5) retrieved from the CMIP website. The climate scenario data was interpolated into 0.5° × 0.5° horizontal resolution and bias-corrected with respect to historical observations to remove systematic errors^46^. The data of maize-planting regions are from the gridded global dataset in 2000 by combining two data products^47,48^.

**Table 1:** Basic information of 5 ESMs in CMIP5.

Model	Research institute	Country	Horizontal resolution
GFDL-ESM2M	Geophysical Fluid Dynamics Laboratory	The United States	144 × 90
HadGEM2-ES	Hadley Center for Climate Prediction and Research	The United Kingdom	192 × 145
IPSL-CM5A-LR	L’ Institute Pierre-Simon Laplace	France	96 × 96
NorESM1-M	Norway Climate Center	Norway	144 × 96
MIROC-ESM	Center for Climate System Research, National Institute for Environmental Studies, and Frontier Research Center for Global Change	Japan	128 × 64

Horizontal resolution means the number of longitudinal grids × the number of latitudinal grids.

### Simulation of climate scenarios with global warming by 1.5 °C and 2.0 °C

**Figure 1:**
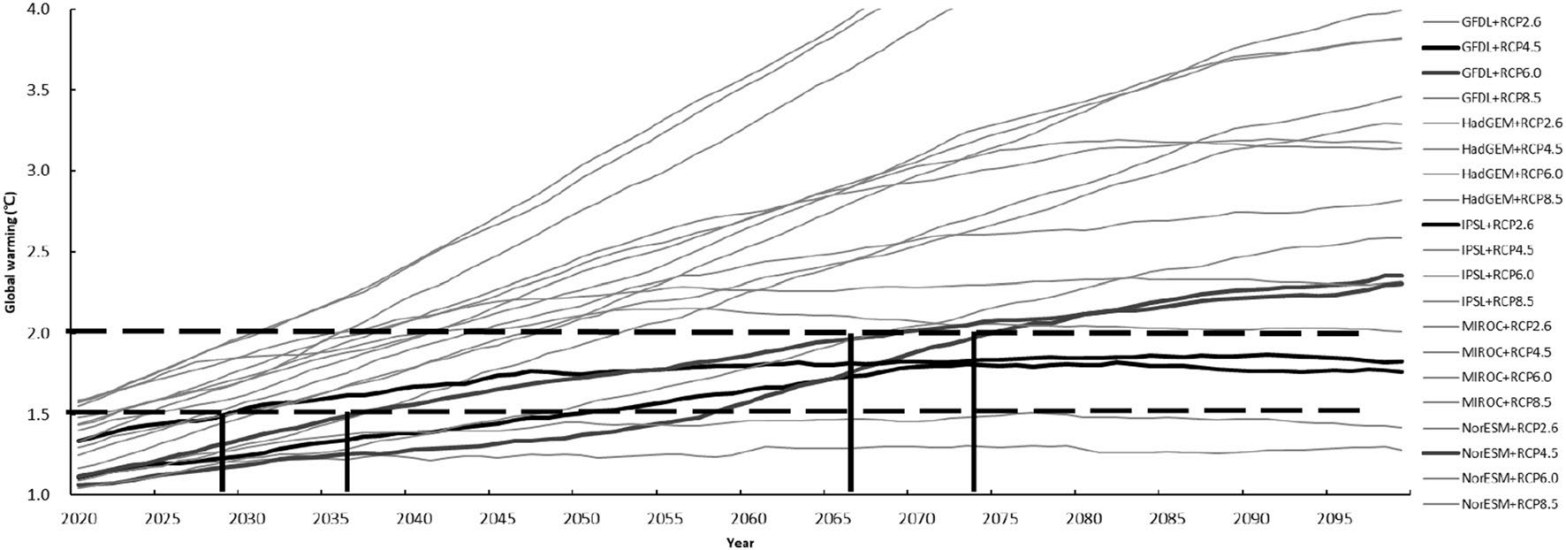
Changes of global temperature of 20 years moving average from 2020 to 2099 simulated by 5 ESMs under 4 RCP scenarios. Note: The black horizontal dashed lines: global warming by 1.5 °C and 2.0 °C; the black vertical solid line: the years when global warming reaches 1.5 °C and 2.0 °C simulated by the selected models and scenarios.

In this study, climate data of global warming by 1.5 °C and 2.0 °C are determined according to the results of global climate models driven by typical concentration paths (RCPs) of greenhouse gas emissions. Eligible data are selected from a total of 20 sets of data under four RCP scenarios of five ESMs (including GFDL-ESM2M, HadGEM2-ES, IPSL-CM5A-LR, MIROC-ESM-CHEM and NorESM1-M), which estimate the temperature, precipitation and sunshine hours (Fig. 1).

Firstly, the period of 1986–2005 is defined as the baseline, of which the simulated average value is recognized as 0.61 °C above pre-industrial (the period of 1850–1900) levels; the baseline is selected according to the accessibility and operability of data, which is used for the determination of the periods with global warming by 1.5 °C and 2.0 °C and the comparison of maize yield between different periods. Secondly, the simulated values of global mean temperature in the future years are subtracted from the simulated average value of 1986–2005; then the values should be plus with 0.61 °C, which are the global warming results above pre-industrial levels; then 20 years moving average of the above results are calculated. Thirdly, the climate data of global warming by 1.5 °C is defined according to the principles provided in the fifth IPCC Assessment Report, for which it should be within 1.5–2.0 °C above pre-industrial levels at the end of the twenty-first century; the climate data of global warming by 2.0 °C is defined according to the principles provided in the fifth IPCC Assessment Report, for which it should be within 2.0–2.5 °C above pre-industrial levels at the end of the twenty-first century and the period of global warming by 2.0 °C should not be earlier than 2050. Finally, the climate models, scenarios and periods of global warming by 1.5 °C and 2.0 °C are separately confirmed; the data of global warming by 1.5 °C, simulated by IPSL-CM5A-LR under RCP2.6 scenario during 2020–2039 and simulated by GFDL-ESM2M under RCP4.5 scenario during 2041–2060; the data of global warming by 2.0 °C, simulated by NorESM1-M under RCP4.5 scenario during 2060–2079 and simulated by GFDL-ESM2M under RCP6.0 scenario during 2065–2084.

### Simulation of maize yield using DSSAT

According to the data of global warming by 1.5 °C and 2.0 °C selected above, we simulated global maize yield changes compared with the average yield during 1986–2005 on grid level using CERES-Maize, which is part of DSSAT version 4.6^49^.

The inputs for DSSAT simulation include daily weather data, soil parameters, crop calendar data and management information. All the inputs are formatted at a 0.5° × 0.5° grid resolution which are computed by high-performance computers. Weather data is from the AgMERRA dataset, including maximum and minimum temperatures, precipitation, total radiation and humidity. Crop calendar data were from the Center for Sustainability and Global Environment (SAGE), in which the existing observations of crop planting and harvesting dates are gridded formatted at a resolution of 5 min^50^. For management information, fertilizer applications, irrigation and other management practices are required. A crop-specific gridded dataset of nitrogen fertilizer application for the world was developed by integrating national and subnational fertilizer application data from a variety of sources, which is used to set up current fertilizer application rates for maize in each grid cell. Soil parameters are from the International Soil Profile Dataset (WISE), including soil texture, bulk density, pH, organic carbon content and fraction of calcium carbonate for each of five 20 cm thick soil layers^51^. All the soil data is allocated to be in accordance with the request of DSSAT simulation; the missing soil parameters for organic soils were adopted from FAO soil dataset.

First maize yields across the world during the historical period 1986–2005 were simulated at the 0.5° × 0.5° grid scale with two main production systems, including Spring maize and Summer maize. Historical national maize production is aggregated from simulated gridded yield and weighted by grid cell maize areas in 2000 from the gridded global dataset by combining two data products^47^. Second, genetic parameters of specific cultivars of maize from previous works were adopted for the initial parameters; model parameters related to crop genotype characteristics were calibrated and tuned following the method in Xiong et al.^52^, in which the simulated yields from 1986–2005 were comparable to the statistical data. Third, maize yields across the world were simulated under global warming by 1.5 °C and 2.0 °C. Finally, global and national maize yields were aggregated from gridded values; changes in national and global yields under global warming by 1.5 °C and 2.0 °C were calculated, comparing maize yield average for 1986–2005.

### Simulation of market price using GTAP

The yield changes for maize from the DSSAT models under 1.5 °C and 2.0 °C temperature increase are used to carry out simulations using competitive market for changes in production, market price, and self-sufficiency ratio of maize at national and global levels^53,54^. For this study, we use a comparative static analysis approach to simulate the impact of climate changes on the prices and trade of the major food crops under current economic conditions. Utilizing current economic conditions has the advantage of minimizing assumptions and model uncertainties related to future economic conditions^55,56^.

The original GTAP database doesn’t include maize as a separate sector, rather it is combined with other coarse grains to form an “other coarse grain” sector. For this study, we updated the GTAP database by splitting maize from the original sector in the database, design an appropriate sectoral and regional aggregation scheme to the original database. The detailed method is given as follows:

First, we improved the database by splitting maize from the existing sector “other coarse grain”, following similar work using GTAP^57–59^ based on the routines from the Splitcom method^60^. In this procedure, the old flows of data both at national and trade levels are allocated between the new flows using weights. The national weights include the division of each unsplit user's use of the original split commodity among the new commodities; the division of unsplit inputs to the original industry between the new industries; the splitting of new industry's use of each new commodity. Maize use is mainly shared between feed, food, processing and others (seed, waste, etc.).

Trade shares allocate the original slice of the split commodity into the new commodity for all elements of basic price value, tax, and margin. Finally, we used the RAS method for balancing the newly created database. The values for the national shares matrix were obtained from FAOSTAT. The trade shares matrix was calculated based on the data from UN Comtrade Database.

Second, our sectoral aggregation scheme for GTAP ensures that all the competing and complimenting sectors for maize are present in the most disaggregated form. For example, for maize, other crops compete for inputs of production and both livestock and households are major users of maize. For regional aggregation, we kept the details for all the main producing, consuming, and trading regions, for maize.

Third, yield shocks for maize were incorporated into the GTAP model via changes in land efficiency for the production of the respective in each region.

## Results

### Climate change under global warming by 1.5 °C and 2.0 °C

**Figure 2:**
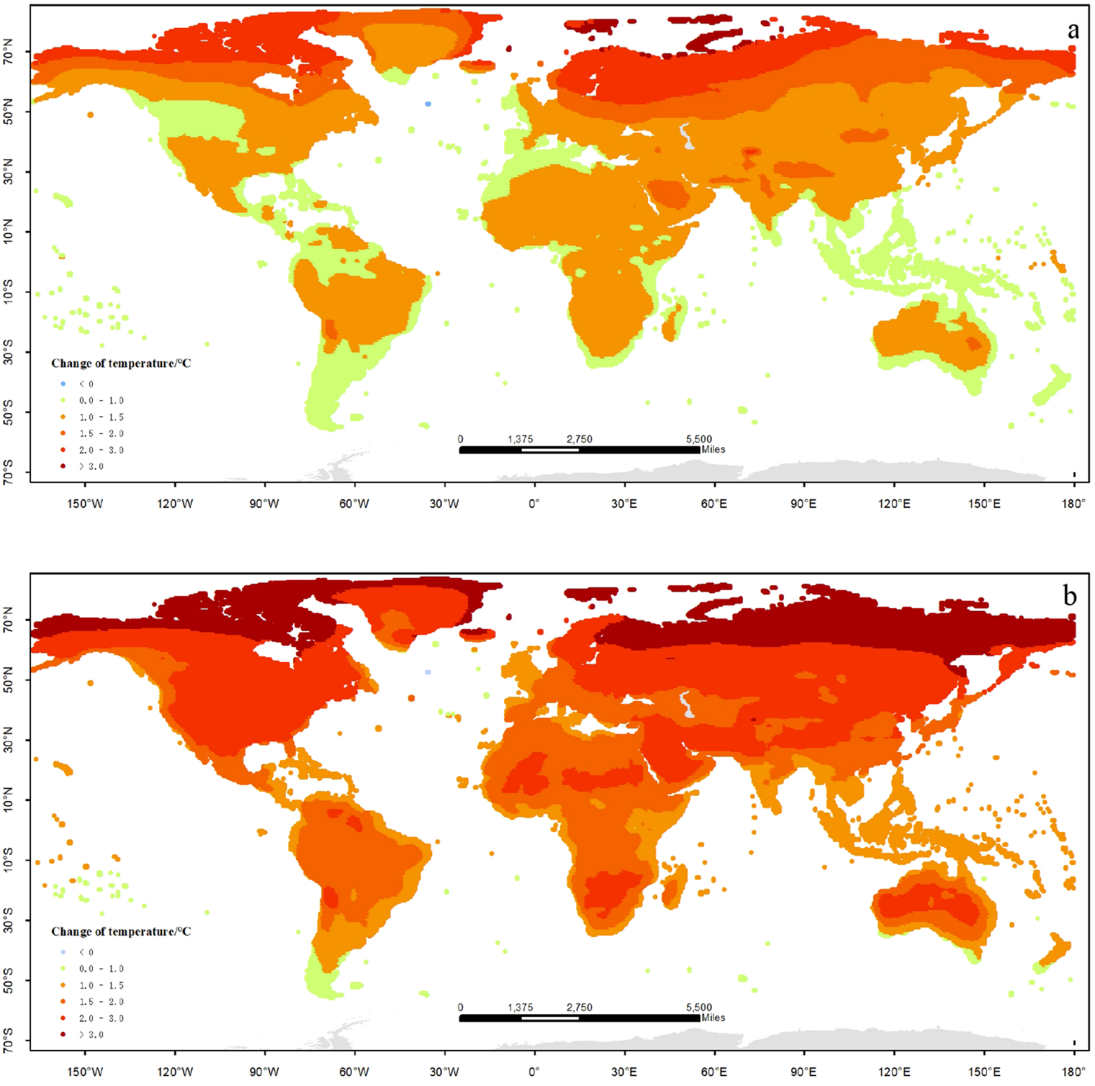

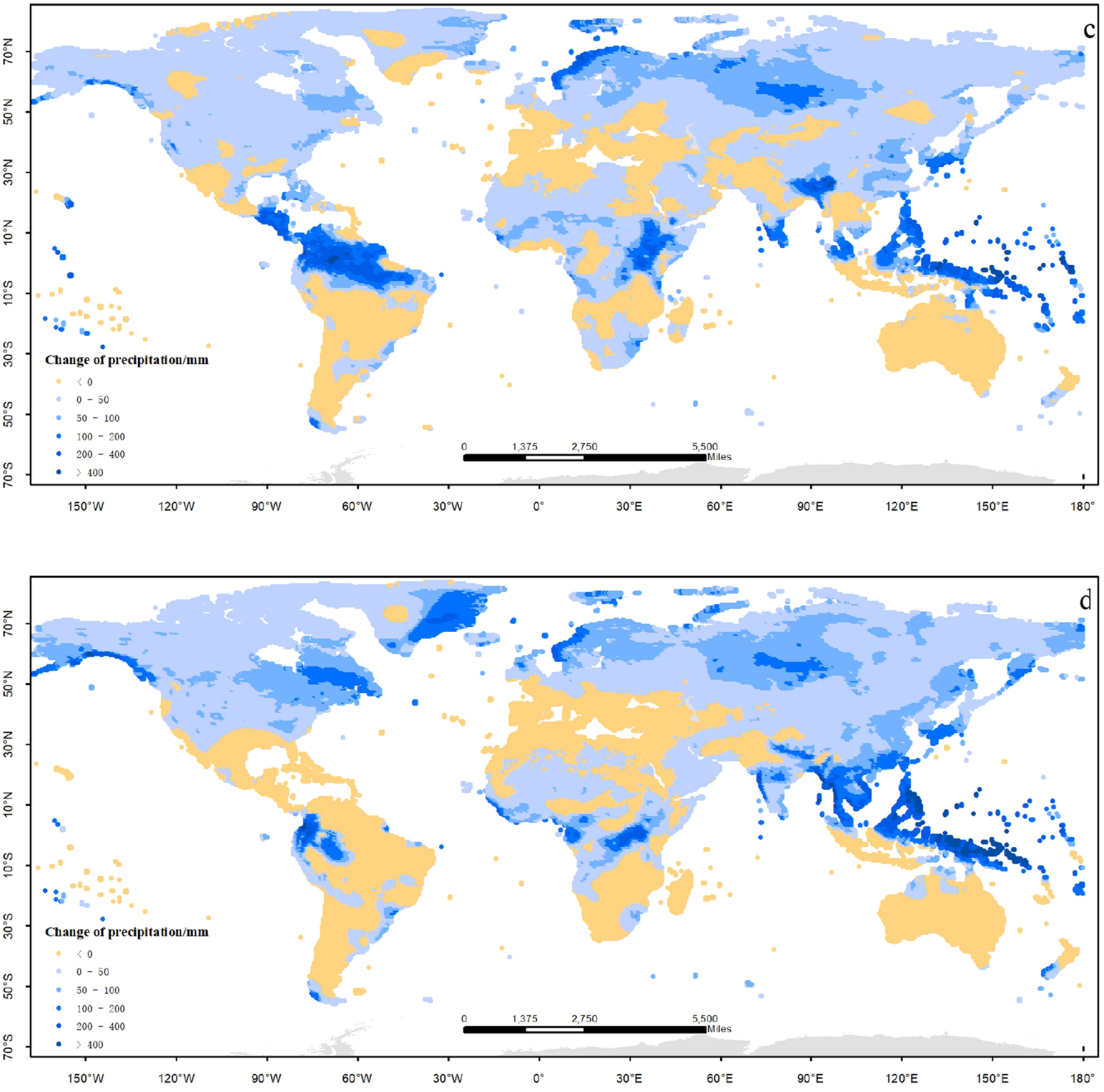
Distribution of temperature and precipitation changes under global warming by 1.5 °C and 2.0 °C (**a**) temperature, 1.5 °C; (**b**) temperature, 2.0 °C; (**c**) precipitation, 1.5 °C; (**d**) precipitation, 2.0 °C. The figure has been generated using ArcGIS 10.2 and Natural Earth-Free vector and raster map data @ https://naturalearthdata.com.

There are apparent change trends of temperature and precipitation relative to the baseline (1986–2005) under global warming by 1.5 °C and 2.0 °C. The most remarkable characteristics is the rising of mean temperature in the worldwide (Fig. 2a, b); meanwhile, the rainfall would increase in most regions of the world. The distributions of temperature changes under global warming by 1.5 °C and 2.0 °C are similar (Fig. 2c, d). There are few regions in which the temperature would go down under the two scenarios; the temperature goes up more seriously in the Northern Hemisphere than the Southern regions; especially in the high-latitude area the temperature rises more quickly than the other regions. Under global warming by 1.5 °C the area is 54.4% in whole world in which the temperature would go up between 1.0 and 1.5 °C than the baseline, located in the middle and low latitude regions; the area is 29.2% of the whole world in which the temperature would go up more than 1.5 °C, most located in the high latitude regions of Northern Hemisphere; the area is 16.4% of the whole world in which the temperature would go up between 0 and 1.0 °C , mostly located in the Southern Hemisphere and the low latitude regions of Northern Hemisphere. Under global warming by 2.0 °C the area is 12.3% in which the temperature would go up between 1.0 and 1.5 °C than the baseline, located in the middle and low latitude regions; the area is 69.8% in which the temperature would go up between 1.5 and 3.0 °C than the baseline, located in the middle and high latitude regions; the area is 16.9% in which the temperature would go up more than 3.0 °C, most located in the high latitude regions of Northern Hemisphere; the area is rarely in which the temperature would go up between 0 and 1.0 °C.

There are apparent trends of humidification in most regions under global warming by 1.5 °C and 2.0 °C; but the drought risk also should be taken seriously in the other regions. Under global warming by 1.5 °C the area is 73.6% of the whole world in which the precipitation would increase, most located in the Northern Hemisphere; the area is 53.7% of the whole world in which the precipitation would increase by less than 50 mm; however, the area is 26.4% of whole world in which the rainfall would decrease, mainly located in the Southern Hemisphere and the middle regions of Northern Hemisphere. The distribution of precipitation under global warming by 2.0 °C is similar with the situation under global warming by 1.5 °C. The drought-threatened area would increase by 28.5% under global warming by 2.0 °C, especially in the middle and low latitude of the Northern Hemisphere; the area would expand to 26%, in which the precipitation increases more than 50 mm. In other words, the extreme rainfall events (such as drought, rainstorm) under global warming by 2.0 °C would be more serious than those under global warming by 1.5 °C, which is what we should be pay more attention to.

### Yield change of maize under global warming by 1.5 °C and 2.0 °C

Maize production is affected by climate change apparently. According to the simulation results of CERES-maize, the yield of maize would decrease in the worldwide relative to 1986–2005 under global warming by 2.0 °C; it would increase little under global warming by 1.5 °C. The distributions of maize yield loss under the two scenarios are similar to each other, mostly located in the middle and low latitude, which are the main regions for maize planting in the world. The loss risk of maize under global warming by 2.0 °C is much more serious than that under global warming of 1.5 °C. However, there are increasing potentials of maize yield in many regions, nearly half of the whole maize planting area in the world, in which the climate situation would become more proper for maize under global warming by 1.5 °C and 2.0 °C. So, there are apparent challenges and opportunities for maize production in the whole world under climate change. We should grasp the opportunities and expand the yield increasing potentials; meanwhile, the threat of maize yield loss should be controlled and compressed to the minimum in the high-risk regions.

**Figure 3:**
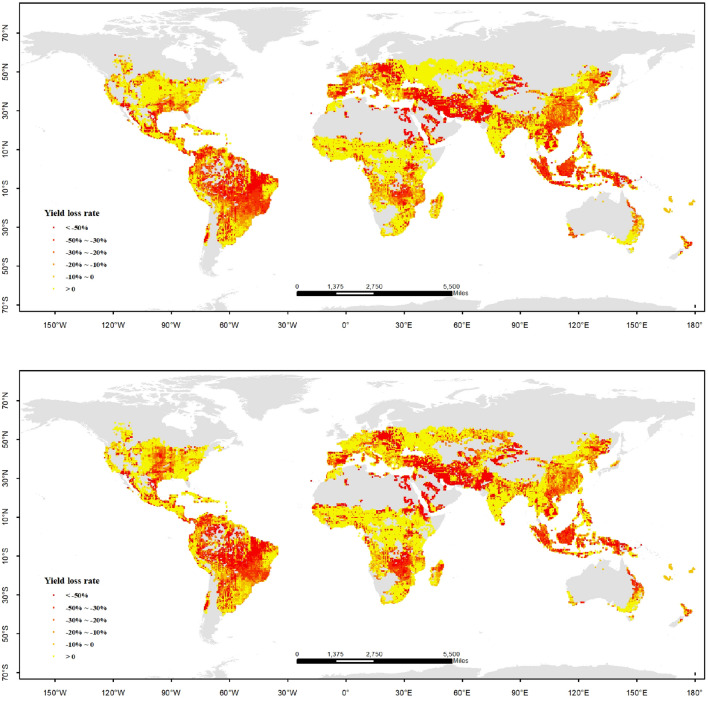
Distribution of yield loss rate on maize in the world under global warming by 1.5 °C (up: IPSL-CM5A-LR model, RCP 2.6; down: GFDL-ESM2M model, RCP 4.5). The figure has been generated using ArcGIS 10.2 and Natural Earth-Free vector and raster map data @ https://naturalearthdata.com.

From the results simulated by IPSL-CM5A-LR model under RCP 2.6 scenario, the gross yield of maize in the world between 2020 and 2039 would decrease by 6.8% relative to 1986–2005. The area is 37.7% of the whole maize planting regions in the world, in which the yield loss would be less than 50%, mainly located in the low and middle latitude of South America and Asia, and the middle latitude of Africa and North America. The area is 16.4% of the whole maize planting regions, in which the yield loss would be more than 50%, mainly located in the low latitude of South America and the middle latitude of Asia and Europe. The area is 45.8% of the whole maize planting regions, in which the yield would increase, mainly located in the low latitude of Africa, Asia and North America, the high latitude of Europe. From the results simulated by the GFDL-ESM2M model under RCP 4.5 scenario, the gross yield of maize in the world between 2041 and 2060 would increase by 7.2% relative to 1986–2005. There are opposite trends of maize yield under global warming by 1.5 °C, which are simulated by different global climate models. However, the spatial distributions of maize yield change are similar to each other. The difference is that the regions of high yield loss rate are decreasing, and the regions of yield increasing are going up. In a comprehensive perspective, under global warming by 1.5 °C, maize yield in the whole world would increase 0.18% relative to 1986–2005 (Fig. 3). According to Paris Agreement, all countries should do their best to limit the global warming by 1.5 °C until the end of 21 century. If that objective could be accomplished, gross maize production of the whole world would not be influenced so much by climate change, but the food security of the whole world would still be attacked violently. There are huge differences among the continents; South America, Asia and the Middle East are threatened seriously by yield loss seriously under global warming by 1.5 °C. The changes in maize yield in different regions would influence the maize price and food trades. So, it should be cautious to cope with the maize changes under global warming by 1.5 °C.

**Figure 4:**
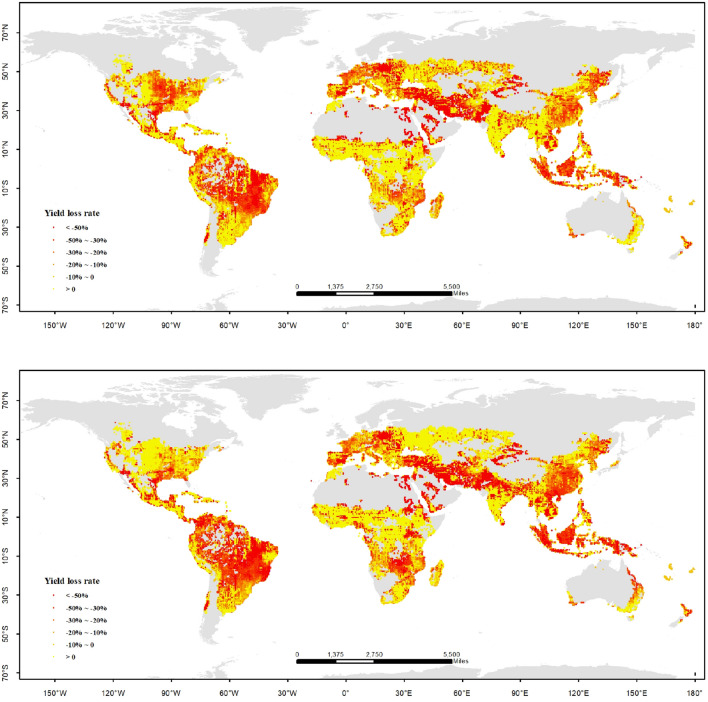
Distribution of yield loss rates on maize in the world under global warming by 2.0 °C (up: NorESM1-M model, RCP 4.5; down: GFDL-ESM2M model, RCP 6.0). The figure has been generated using ArcGIS 10.2 and Natural Earth-Free vector and raster map data @ https://naturalearthdata.com.

From the results of simulated by the NorESM1-M model under RCP 4.5 scenario, the gross yield of maize in the world between 2060 and 2079 would decrease by 18.7% relative to 1986–2005. The area is 41.7% of the whole maize planting regions in the world, in which the yield loss would be less than 50%. The area is 15.6% of the whole maize planting regions, in which the yield loss would be more than 50%. The area is 42.7% of the whole maize planting regions, in which the yield would increase. The distribution of maize yield change is similar to that under global warming by 1.5 °C. From the results simulated by the GFDL-ESM2M model under RCP 6.0 scenario, the gross yield of maize in the world between 2065 and 2084 would decrease by 3% relative to 1986–2005. Comparing to the results of the NorESM1-M model, the regions of high yield loss rate are increasing, and the regions of yield increases are going down; but the per unit area yields are increasing quickly in the regions of yield increasing. So, the gross maize yield in the whole world simulated by the GFDL-ESM2M model is more than the NorESM1-M model. In a comprehensive perspective, under global warming by 2.0 °C, maize yield in the whole world would decrease 10.8% relative to 1986–2005 (Fig. 4). Compared to the results under global warming by 1.5 °C, the risk of yield loss is much higher. According to the new results from the Emission Gap Report in 2019, the target of global warming by 1.5 °C would not be implemented according to the reality of mitigation actions; the chance become much bigger for all countries in the world, who will be facing the severe challenge of global temperature rise of 2.0 °C or even higher (3.0 °C or 4.0 °C) in the future. So it is critical to cope with the serious condition that maize yield would decrease severely. For the whole world more mitigation and adaptation actions should be taken from now on. Food security would be a significant challenge in this century.

### Yield change of maize in main countries

**Figure 5:**
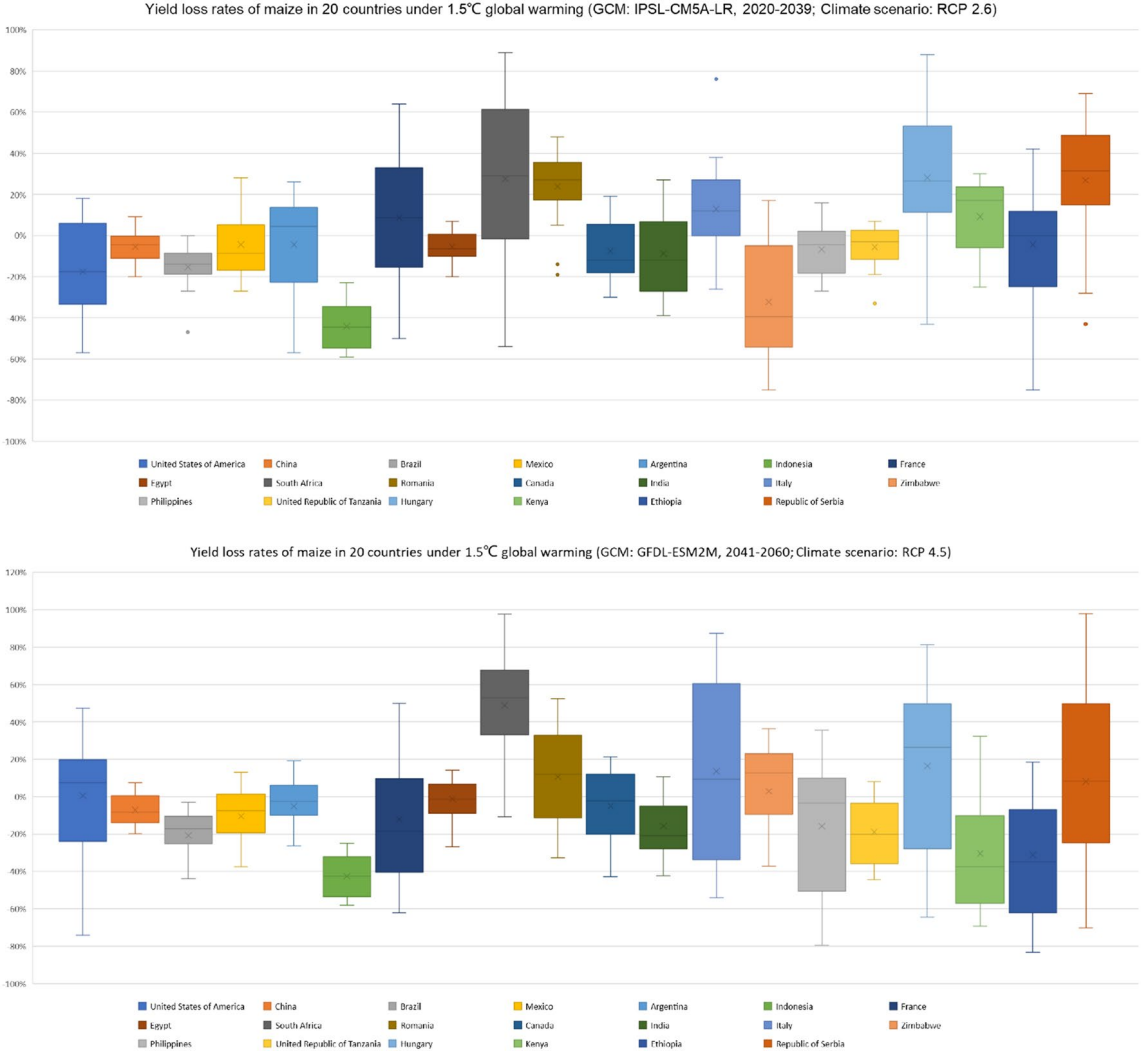

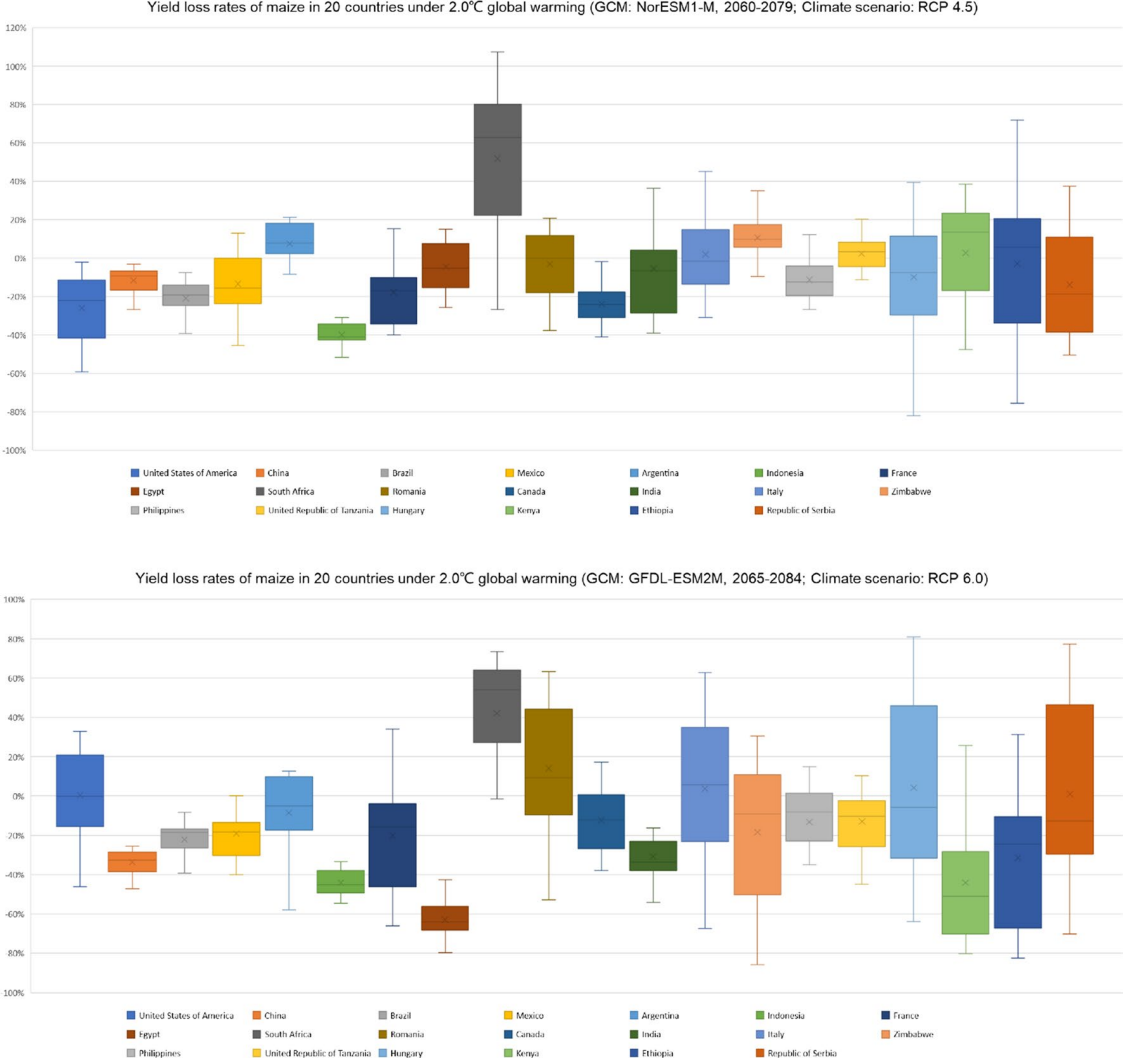
Yield loss rates on maize in top 20 countries under global warming by 1.5 °C and 2.0 °C.

There are huge differences in impacts on maize yield under climate change, which would influence the food crisis in different regions. There are 159 countries in the whole world which plant maize. The gross yield of maize the top 20 countries accounts for more than 90% of the total yield in the 159 countries. So, the changes in the top 20 countries under future scenarios would influence the food security of the whole world (Fig. 5). From the results of simulated by CRESE-maize under global warming by 1.5 °C, there would be 75 countries facing with yield loss of maize; the mean yield loss rate would become 33.5%. There would be 84 countries experiencing yield increases. Overall, the global maize yield would slightly increase. Under global warming by 2.0 °C, there would be 82 countries facing with yield loss of maize, for which the mean yield loss rate is approximate to that under global warming by 1.5 °C. There would be 77 countries experiencing yield increase; however, the mean yield increase is apparently smaller than that under global warming by 1.5 °C. Generally, the global maize yield would decrease. The results show that the adverse effect of warming up 2.0 °C on global maize production is far greater than warming up 1.5 °C. It is important to take actions to develop forward-looking adaptation measures to cope with future climate change.

According to statistics in 2018, the gross maize yield in the top 5 countries is almost 80% of the total maize yield of the whole world. The United States accounts for more than 32%; China accounts for about 24%; Brazil, Argentina and Mexico account for about 23%. The fluctuation of maize production in these five top countries will have a significant impact on the global maize trade. Based on the simulation results, comparing to 1986–2005, the maize yield in China, Brazil and Argentina would decrease under global warming by 1.5 °C; the yield loss rate would reach more than 20% in Brazil; Argentina would decrease by 14.7%; China would decrease by 3.7%. However, there would be increasing trends in the United States and Mexico; the change in the United States would not be significant and the maize yield would increase by 0.5%; the yield increasing rate would exceed 50% in Mexico. Overall, the gross maize yield in the top 5 countries would decrease by 2% under global warming by 1.5 °C. According to the simulation results, comparing to 1986–2005, the maize yield in the United States, China and Brazil would decrease under global warming by 2.0 °C; the yield loss rate would reach more than 24% in Brazil; the United States would decrease by 13.3%; China would decrease by 11.5%. However, there would be increasing trends in Argentina and Mexico; the maize yield would increase by 16.8% in Argentina; the yield increasing rate would exceed 40% in Mexico. Overall, the gross maize yield in the top 5 countries would decrease by 11.4% under global warming by 2.0 °C. By comparing the maize production in different countries, it can be found that the reduction trend of total maize production in the top five countries is more obvious, especially under the scenario of global warming by 2.0 °C, the global food trade and food security may face greater risks.

**Figure 6:**
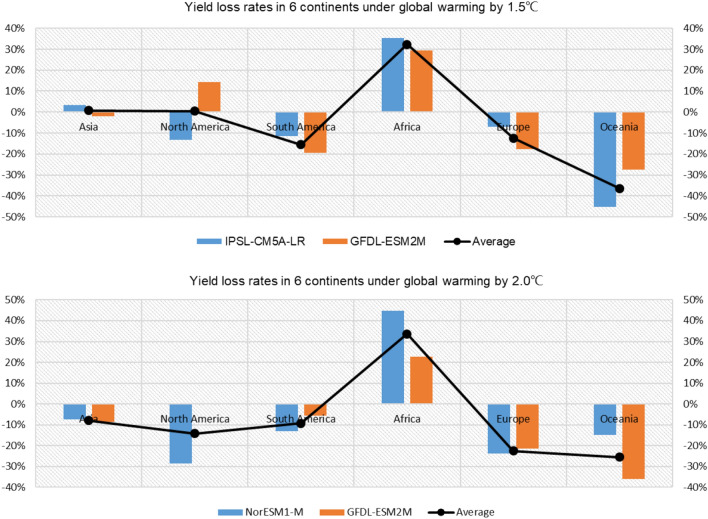
Yield loss rates on maize in 6 continents under global warming by 1.5 °C and 2.0 °C.

From the view of continents, there are different trends of maize yield changes in the 6 continents (except Antarctica) under global warming by 1.5 °C and 2.0 °C (Fig. 6). From the results of simulated by CRESE-maize under global warming by 1.5 °C, the maize yield in 3 continents would decline apparently, including South America, Europe and Oceania; the average yield loss rates are respectively − 15.6%, − 12.4%, − 36.4%; in the other 3 continents the average maize yield would go up, especially in Africa more than 30%; the increasing trends are slight in Asia and North America, in which the yield increasing rates are separately 0.7% and 0.4%. However, the yield change trends simulated by IPSL-CM5A-LR and GFDL-ESM2M models are different in 2 continents, including Asia and North America. From the results of simulated by CRESE-maize under global warming by 2.0 °C, the maize yield in 5 continents would decline apparently, except Africa; the average yield loss rates are respectively − 7.9% (Asia), − 14.1% (North America), − 9.3% (South America), − 22.5% (Europe), − 25.5% (Oceania); only in Africa the average maize yield would go up also more than 30%; meanwhile the yield change trends simulated by IPSL-CM5A-LR and GFDL-ESM2M models are the same in each continent. Comparing the two global warming scenarios, there would be apparent variations in maize yield in Asia and North America, in which the annual maize yield accounts for a great proportion of the whole world, leading to a much more serious yield loss under global warming by 2.0 °C than that under global warming by 1.5 °C. There would be an obvious crisis of food supply under global warming by 2.0 °C with the increasing population in the future. So, it is important to make full preparation for adaptation to climate change in the whole world.

### Market price of maize in main countries

In this study, we elaborate on the endogenous response of our economic models. This response can be theoretically elaborated as: due to the effect of climate change on yield reduction (improvement), the supply curve moves leftward (rightward), reducing (increasing) production and raising (lowering) prices. In response, the consumers decrease (increase) their consumption of more expensive (cheaper) crops and shifting to other (increase the use of the same) crops. Producers, at the same time, respond by changing farm-level management practices and increasing (decreasing) the amount of acreage under these crops. At a global scale, the reallocation of production and consumption through international trade further alters climate change impacts on global agriculture. This also alters the self-sufficiency ratios of each country/region due to climate change.

**Figure 7:**
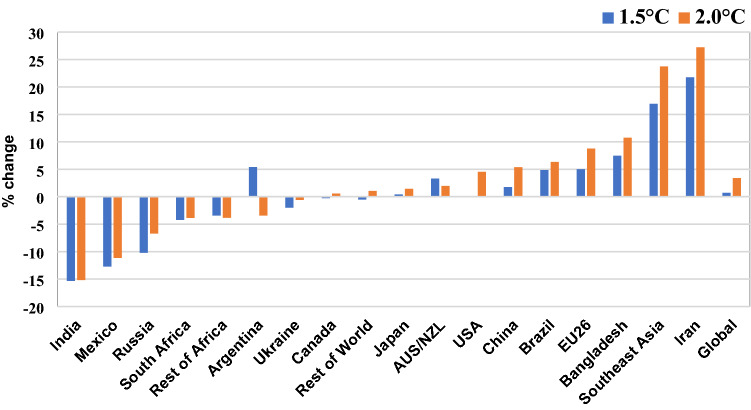
Price change on maize in main continents under global warming by 1.5 °C and 2.0 °C.

**Figure 8:**
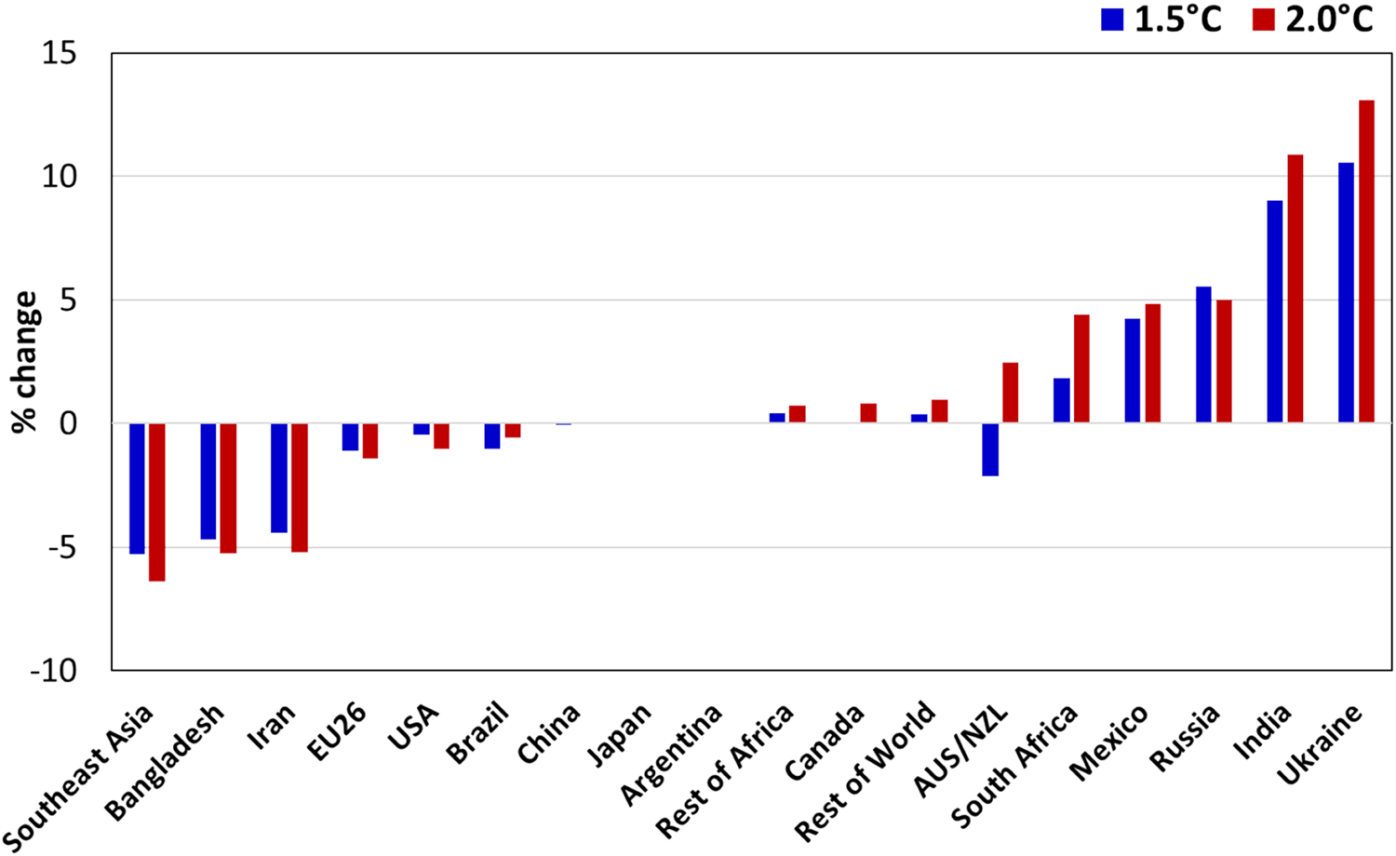
Changes in Self-sufficiency ratio of maize in main countries under global warming by 1.5 °C and 2.0 °C.

In response to production changes, the price of each commodity changes under both scenarios. At the global level, the market price for maize would increase by 0.7% and 3.4% under 1.5 °C scenario and 2.0 °C scenario, respectively, which would vary quite largely among different countries and regions under both climate change scenarios (Fig. 7). Particularly, the market price would increase by around 22% and 27% in Iran under 2.0 °C scenario and 1.5 °C scenario, respectively. Iran is also the region where the highest yield reduction is observed due to climate change. Market prices for maize in India, Mexico, Russia, South Africa and the Rest of Africa would decrease significantly under both scenarios, as their yields improve due to climate effects. Along with the domestic production, the climate change will also induce changes in international trade of maize, resulting in changing levels of self-sufficiency ratios (SSR) for each country/region. By SSR, we mean the ratio of domestically produced commodity, to the sum of net imports and domestic production. In our scenario analysis, generally, the countries that face positive effects on yields and/or are relatively less dependent on imports, are positively (less negatively) affected by climate change. For example, maize SSR for Ukraine, India, Russia and Mexico would improve under both scenarios (Fig. 8). Whereas the self-sufficiency ratios of maize for Southeast Asia, Bangladesh and Iran will worsen under both scenarios. China’s SSR for maize stays almost similar to the level as the baseline.

## Discussion and conclusion

### Discussion

Our analysis highlights the effects of climate change on global- and regional-specific maize yields and the associated economic consequences in 1.5 °C and 2.0 °C -warming scenarios. We find that the reduction risk of maize yield under global warming by 2.0 °C is much more serious than that under global warming by 1.5 °C. On the one hand, the larger the temperature rise, the greater the evapotranspiration would be. Although the precipitation is also increasing, the evapotranspiration would become more intense. The limitation of water supply for maize growth leads to the decline of yield. On the other hand, relative to global warming by 1.5 °C, maize production would be faced with more serious and frequent extreme climate events, such as drought and heat waves, which would increase the risk of corn yield reduction under global warming by 2.0 °C. In the meantime, the huge differences in yield changes in different regions provide a small chance for the world, especially under global warming by 1.5 °C. In the near future, if the global temperature can be effectively controlled under 1.5 °C warming scenario, there would be an increase in the potential for maize yield in the worldwide. All regions and countries should take actions to reduce the yield loss risk. For the yield-increasing regions, the potentials of climate resources should be fully utilized to guarantee maize yield under future scenarios; for the yield-reducing regions, the targeted adaptation actions should be taken in advance under global warming by 1.5 °C and 2.0 °C.

Meanwhile, the risk of price fluctuations caused by global corn trade due to future climate change should be paid more attention to, especially for developing and undeveloped countries. In the view of supply and demand, the population would go up quickly in the next 30 years; the demand for maize would increase hugely; however, the supply of maize would go down in the future, especially under global warming by 2.0 °C; it would intensify the contradiction between supply and demand, which would threaten the food security and sustainable development in the whole world.

In this study, 5 climate models are selected, which are recommended by ISI-MIP (The Inter-Sectoral Impact Model Intercomparison Project); compared with other climate models, the five models could more effectively support impact assessment in different sectors and provide more reliable results. Based on the simulation results from 5 climate models under 4 RCP scenarios, the future climate situations were selected which are the approximate scenarios with global warming by 1.5 °C and 2.0 °C at the end of 21 century relative to pre-industrial levels; it could minimize the uncertainties of future climate data. The inputs for DSSAT simulation include soil parameters, crop calendar data and management information are coped with carefully to improve the effectiveness and reliability of maize yield simulation.

There are also several uncertainties and limitations. Firstly, there is no unified understanding of how to calculate the temperature rise of 1.5 °C and 2.0 °C relative to pre-industrial levels in the worldwide. At present the research on climate prediction and impact assessment under global warming 1.5 °C and 2.0 °C usually adopts multi-mode ensemble average methods^61,62^, which could obtain the warming response under the condition of instantaneous change, rather than the warming process under the stable state expected by the long-term goal. If we expect to obtain the accurate results, the model prediction test should be estimated to form proprietary scenarios for global warming by 1.5 °C and 2.0 °C^63,64^, which could support for the impacts assessment on different sectors. Some institutions are carrying out climate change predictions under the lower emission scenarios (global warming 1.5 °C or 2.0 °C). At the same time, in order to achieve the goal of controlling temperature by 1.5 °C at the end of the twenty-first century, it is urgent to take actions to reduce emissions and develop along the track of low energy consumption^65,66^; but it is a great challenge for human society to achieve this goal.

Secondly, our methodological approach in this study also has some important limitations, including our use of a single crop model to estimate maize yields. There are some limitations for the DSSAT model to simulate yield loss caused by climate extreme events^67^, in which the impacts of pests and diseases are also ignored^68^. However, the DSSAT model has been applied in a lot of researches to simulate historical maize yield^69–71^, in which the results are reliable and credible^72^. The results of this research could be an important reference to the other studies which simulate global maize yield in the future, applying crop models such as APSIM, WOFOST, ORYZA and so on.

Thirdly, there are relatively more researches on the prediction of climate change trend under the background of 1.5 °C and 2.0 °C; but the research on the impact assessment of the main grain crops including global trade in worldwide is few. In the meantime, we do not assess the effect of future changes on agriculture, such as increases in farm productivity due to new technology. The maize planting area in the future is assumed to be the same as the current situation of maize cultivation in the world.

### Conclusion

According to the simulation results, the yield of maize under global warming by 2.0 °C would decrease between 3.0 and 18.7% in the worldwide relative to 1986–2005; the maize yield would fluctuate between − 6.8 and 7.2% under global warming by 1.5 °C. From the spatial distribution, the gross maize yield in the top 5 high-yield countries (including the United States, China, Brazil, Argentina and Mexico) would decrease by 2% under global warming by 1.5 °C and 11.4% under global warming by 2.0 °C. At the global level, the market price for maize would increase by 0.7% and 3.4% under 1.5 °C scenario and 2.0 °C scenario, respectively, which would vary quite largely among different countries and regions. So, it is urgent for all countries to pay enough attention to the loss risk of maize yield and take actions of mitigation and adaptation to climate change. The time left for changing our minds and actions is becoming less and less.

## Data Availability

The historical weather data (1986–2005) that support the analysis with ESMs in this study are publicly available online at https://data.giss.nasa.gov/impacts/agmipcf/; the future climate scenario data (2006–2099) that support the analysis with ESMs in this study are publicly available online at https://pcmdi.llnl.gov/?cmip5 and https://esgf-node.llnl.gov/projects/esgf-llnl/. The spatial data of harvest area, yield, crop calendar, irrigation portion and chemical N input for maize that support the simulation with crop model (DSSAT) in this study are publicly available at http://mapspam.info/ (SPAM) and http://www.sage.wisc.edu (SAGE); the soil data that support the simulation with crop model (DSSAT) in this study are publicly available from the WISE database (https://www.isric.online/index.php/) and the Digital Soil Map of the World (DSMW) (http://www.fao.org/land-water/land/land-governance/land-resources-planning-toolbox/category/details/en/c/1026564/). All other relevant data are available from the corresponding authors.
